# Gene Therapy Strategy for Alzheimer’s and Parkinson’s Diseases Aimed at Preventing the Formation of Neurotoxic Oligomers in SH-SY5Y Cells

**DOI:** 10.3390/ijms222111550

**Published:** 2021-10-26

**Authors:** Assou El-Battari, Léa Rodriguez, Henri Chahinian, Olivier Delézay, Jacques Fantini, Nouara Yahi, Coralie Di Scala

**Affiliations:** 1 INSERM UMR_S 1072, Aix-Marseille Université, 13015 Marseille, France; assou.elbattari@wanadoo.fr (A.E.-B.); henrichahinian@gmail.com (H.C.); jm.fantini@gmail.com (J.F.); nouara.y@gmail.com (N.Y.); 2 CUO-Recherche, Département d’ophtalmologie, Faculté de Médecine, Université Laval and Centre de recherche du CHU de Québec-Université Laval, Québec, QC G1V 0A6, Canada; lea.rodriguez.1@ulaval.ca; 3 Faculté de Médecine, SAINBIOSE INSERM U1059, Campus Santé Innovations, 42270 St. Priest en Jarez, France; olivier.delezay@univ-st-etienne.fr; 4 Neuroscience Center—HiLIFE, Helsinki Institute of Life Science, University of Helsinki, 00014 Helsinki, Finland

**Keywords:** amyloid proteins, oligomeric pores, calcium, gangliosides

## Abstract

We present here a gene therapy approach aimed at preventing the formation of Ca^2+^-permeable amyloid pore oligomers that are considered as the most neurotoxic structures in both Alzheimer’s and Parkinson’s diseases. Our study is based on the design of a small peptide inhibitor (AmyP53) that combines the ganglioside recognition properties of the β-amyloid peptide (Aβ, Alzheimer) and α-synuclein (α-syn, Parkinson). As gangliosides mediate the initial binding step of these amyloid proteins to lipid rafts of the brain cell membranes, AmyP53 blocks, at the earliest step, the Ca^2+^ cascade that leads to neurodegeneration. Using a lentivirus vector, we genetically modified brain cells to express the therapeutic coding sequence of AmyP53 in a secreted form, rendering these cells totally resistant to oligomer formation by either Aβ or α-syn. This protection was specific, as control mCherry-transfected cells remained fully sensitive to these oligomers. AmyP53 was secreted at therapeutic concentrations in the supernatant of cultured cells, so that the therapy was effective for both transfected cells and their neighbors. This study is the first to demonstrate that a unique gene therapy approach aimed at preventing the formation of neurotoxic oligomers by targeting brain gangliosides may be considered for the treatment of two major neurodegenerative disorders, Alzheimer’s and Parkinson’s diseases.

## 1. Introduction

Neurological disorders, including Alzheimer’s and Parkinson’s diseases (AD and PD, respectively), are currently the leading source of disability around the world, and their prevalence will continue to grow exponentially as the population ages [1,2]. Current treatments are limited to relieving the symptoms of these diseases; however, with various complications leading to severe side effects [3,4]. It is therefore urgent to find an efficient treatment targeting the underlying disease mechanisms. For decades, insoluble aggregates of amyloid proteins have been considered as the main culprits in AD and PD [5]. However, this notion has now been dismissed and, instead of these large aggregates, small oligomers of amyloid proteins, including β-amyloid peptide (Aβ) and α-synuclein (α-syn), are now considered as the primary neurotoxic species in AD and PD [6,7,8,9,10,11]. Indeed, there are several documented cases of old people without any typical neurological symptom yet displaying abundant senile plaques in their brain [12,13,14,15]. Correspondingly, most therapeutic approaches to AD based on the clearing of amyloid plaques have failed [16], and we are now looking for alternative strategies targeting neurotoxic oligomers for both AD and PD [17,18,19,20,21].

Among these oligomers, those forming amyloid pores in the plasma membrane of brain cells are highly neurotoxic since they allow a massive and non-reversible entry of Ca^2+^ ions [22,23,24,25]. Our group has previously identified the molecular mechanisms controlling the formation of Aβ and α-syn pores in lipid rafts of human brain cells [26,27,28]. We identified gangliosides as the primary target of any amyloid proteins on neurons and astrocytes and determined which part of the protein is involved in ganglioside recognition, e.g., domain 5–16 of Aβ and domain 34–45 of α-syn [29,30]. A detailed analysis of the involvement of gangliosides in the toxicity of amyloid oligomers has recently confirmed that these membrane glycolipids are a relevant therapeutic target for both AD [19] and PD [31]. We then designed a chimeric α-syn/Aβ peptide (AmyP53) mixing the ganglioside-binding domain of both proteins to improve ganglioside recognition [30].

To test the therapeutic potential of AmyP53, we developed a sensitive amyloid pore assay based on the detection of Ca^2+^ fluxes induced by amyloid pore oligomers generated in the minutes following the addition of nanomolar concentrations of Aβ1–42 or α-syn to recipient SH-SY5Y cells [32,33]. Using this assay, we demonstrated that AmyP53 prevents the formation of such amyloid pores (both wild type Aβ and α-syn, but also mutated forms including A30P, E46K and A53T that are associated with inherited forms of PD) [32].

Parallel strategies aimed at blocking amyloid pore formation have recently led to the design of a series of active small compounds and antibodies [19]. However, such solutions are hampered by several issues such as solubility, specificity, high concentrations required and potential toxicity [19]. These pitfalls are usually not encountered with therapeutic peptides, which are generally well tolerated because they are specific by design and active at low concentrations [34]. Moreover, a therapeutic peptide can be chronically synthesized by recipient cells following the transfer of a synthetic gene encoding its amino acid sequence [35,36]. Indeed, several gene therapies are currently under evaluation for AD [37] and PD [38], yet they are generally focused on neuroprotection (e.g., [36,39,40]). In the case of AmyP53, such a gene therapy would directly tackle the root cause of the disease, i.e., oligomer formation in the plasma membrane of brain cells [13,18,19,31,41,42]. In the present study, we evaluated the therapeutic potential of AmyP53 in human brain cells after transfection with a lentiviral vector. We show that the genetic transfer of the AmyP53 sequence is remarkably efficient and induces the chronic biosynthesis and secretion of the therapeutic peptide without any toxicity. Most importantly, we show that the recipient cells become resistant to amyloid pore formation by both α-syn and Aβ.

## 2. Results

### 2.1. Engineering the Transgenic Cell Line That Constitutively Secretes AmyP53

**Figure 1:**
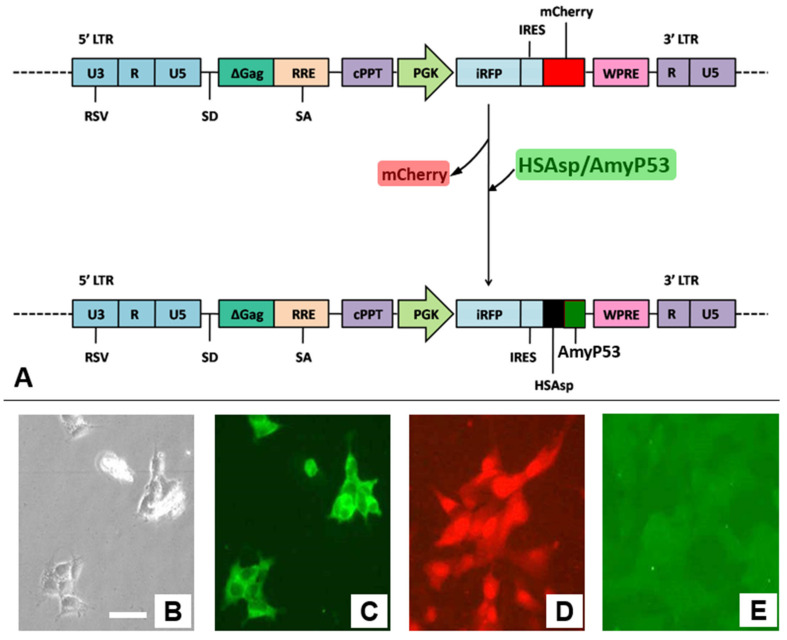
Specific detection of AmyP53 peptide in transfected cells. (**A**) Genetic construction used for engineering SH-SY5Y-AmyP53 cells and control SH-SY5Y-mCherry cells. (**B**) Phase contrast micrograph of SH-SY5Y-AmyP53 cells. (**C**) Cell surface immuno-labeling of SH-SY5Y-AmyP53 with the anti-AmyP53 peptide antibody (same field as in panel **B**). (**D**) mCherry fluorescence of SH-SY5Y-mCherry cells. (**E**) Absence of detection of AmyP53 in control SH-SY5Y-mCherry cells. Scale bar: 25 µm.

Given the unique mechanism of action of AmyP53 and considering that its amino acid sequence can be encoded genetically, we evaluated the possibility of engineering a transgenic cell line that would chronically secrete the peptide. To this end, we constructed a retroviral vector in which the nucleotide coding sequence of AmyP53 was ligated with a signal peptide encoding motif, as detailed in Materials and Methods (Figure 1A). This vector was used to transfect human neuronal SH-SY5Y cells, which are highly sensitive to amyloid oligomer neurotoxicity [28,32]. The resulting transfected cell line, referred to as SH-SY5Y-AmyP53, could be maintained in cell culture under standard conditions (Figure 1B). These cells were homogenously labeled with anti-AmyP53 antibodies (Figure 1C).

The control of transfection was performed with the same retroviral vector in which the AmyP53 coding sequence was replaced by the one coding for the red fluorescent protein mCherry, leading to similar transfection yields (Figure 1D). As expected, these transfected cells were not labeled by anti-AmyP53 antibodies (Figure 1E).

The secretion of AmyP53 by SH-SY5Y-AmyP53 cells was assessed by an ELISA method carried out on cell culture media collected at different times (Figure S1). A mean concentration of 2.1 µg/mL/24 h (1.53 µM/24 h) was measured, corresponding to a kinetic of 87 ng/mL/h (64 nM/h). The supernatant of control cells did not contain detectable AmyP53 (Figure S1).

To assess whether the concentration of AmyP53 secreted by SH-SY5Y-AmyP53 cells could block the formation of oligomeric pores under physiologically relevant conditions, we determined the effective 50% concentration (EC_50_) of AmyP53 dose. In this dose-effect study, non-transfected SH-SY5Y were preloaded with Fluo-4AM and then probed with 200 nM of Aβ1–42 in the presence of various concentrations of AmyP53. As shown in Figure S2, AmyP53 induced a dose-dependent inhibition of Ca^2+^ entry through neurotoxic Aβ1–42 oligomers, with an effective 50% concentration (EC_50_) of 30 nM. Thus, the concentration of AmyP53 produced by SH-SY5Y-AmyP53 cells (64 nM/h) is consistent with a lifetime protection against the neurotoxicity of amyloid oligomers. To confirm these theoretical calculations, we studied the sensitivity of SH-SY5Y-AmyP53 cells to the neurotoxicity of Aβ1–42 and α-syn oligomers.

### 2.2. SH-SY5Y-AmyP53 Cells Are Constitutively Protected from Amyloid Oligomer Formation

**Figure 2:**
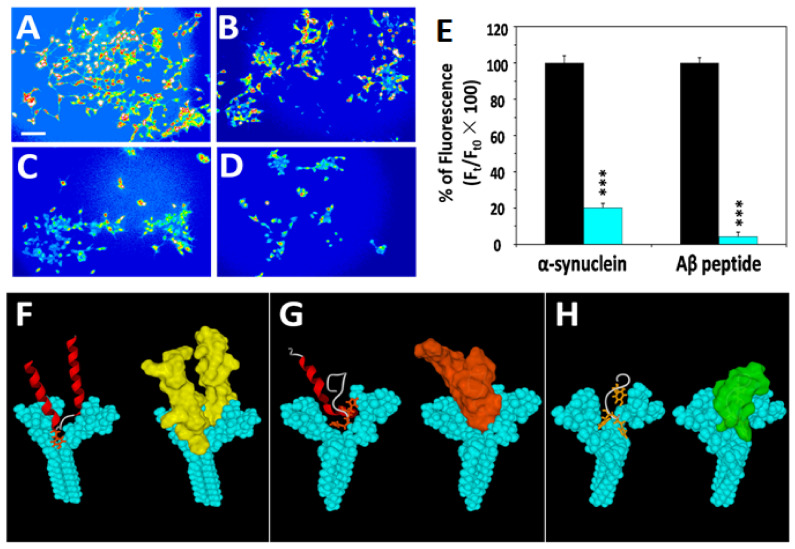
Resistance of transfected cells to amyloid pore formation by α-syn and Aβ. (**A**,**B**) Non-transfected SH-SY5Y cells. (**C**) SH-SY5Y-AmyP53 cells incubated with Aβ1–42. (**D**) SH-SY5Y-AmyP53 cells incubated with α-syn. In these experiments, the cells were first loaded with Fluo-4AM and Ca^2+^-dependent fluorescence was measured 60 min after injection of Aβ1–42 peptide (**A**,**C**), or 75 min after injection of α-syn (**B**,**D**) at a concentration of 220 nM. The images show pseudocolor representations of cells (scale bar: 100 µm), with warmer colors corresponding to a higher fluorescence. The pictures are representative of three independent experiments. (**E**) Results in the histogram are expressed as mean ± SEM. Student’s *t*-test was used to compare the statistical significance of fluorescence between non-transfected and SH-SY5Y-AmyP53 cells + α-syn (*** *p* < 0.0005 with 52 < *n* < 95) or + Aβ (*** *p* < 0.0005 with 111 < *n* < 151). (**F**–**H**) Molecular modeling of ganglioside-α-syn (**F**), ganglioside-Aβ (**G**) and ganglioside-AmyP53 (**H**) complexes. In each case, two views of the molecular complex are shown, with the ganglioside dimer colored in cyan and the ligand (α-syn, Aβ1–42 or AmyP53) in secondary structure (**left panels**) and surface (**right panels**) rendition. The lateral chains of tyrosine (**F**) and histidine (**G**,**H**) residues are represented. These amino acids are deeply inserted in the chalice formed by the dimer of gangliosides. In addition, note how the pair of histidine residues of AmyP53 (H13–H14) clamps the peptide on the ganglioside dimer, resulting in tighter interaction compared with α-syn or Aβ. The third histidine residue of AmyP53 interacts with the glycone part of the ganglioside on the right side of the dimer.

In these experiments, SH-SY5Y-AmyP53 cells were preloaded with Fluo-4AM, then rinsed and further incubated in a buffer containing 200 nM of either Aβ1–42 or α-syn. At this stage, the initial culture supernatants of AmyP53-producing cells were no longer present in the incubation media. Under these conditions, only AmyP53 peptides already bound to the plasma membrane of the cells may still be present, in agreement with the immunodetection of AmyP53 on the cell surface of SH-SY5Y-AmyP53 cells (Figure 1C). As shown in Figure 2, the typical Ca^2+^ fluxes through Aβ1–42 or α-syn oligomeric pores could not be observed (or were dramatically reduced) in SH-SY5Y-AmyP53 cells. These data indicated that SH-SY5Y-AmyP53 cells were indeed protected from the formation and thus the neurotoxicity of Aβ1–42 and α-syn oligomers.

Most importantly, control mCherry-transfected cells (Figure S3) remained fully sensitive to neurotoxic oligomers. Thus, the protective effect observed in Figure 2 could be attributed to the selective anti-oligomer activity of AmyP53 produced by SH-SY5Y-AmyP53 cells.

**Figure 3:**
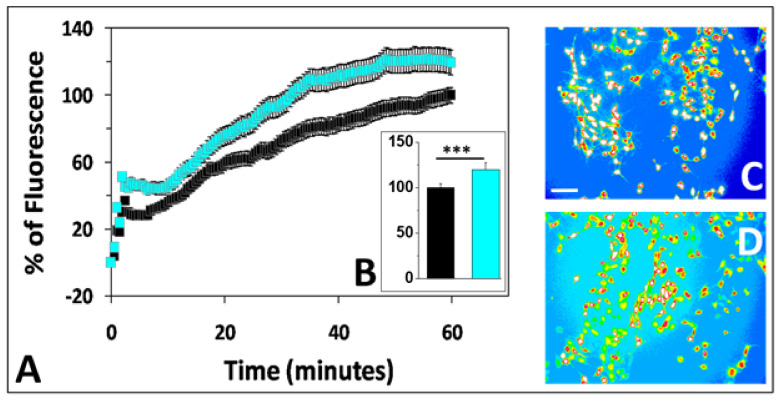
Ca^2+^ entry into SH-SY5Y-AmyP53 cells treated with the calcium ionophore A23187.SH-SY5Y-AmyP53 cells were preloaded with Fluo-4AM, then incubated with A23187 at a concentration of 0.2 µM (black symbols) or 2 µM (cyan symbols). The data show the kinetics (**A**), histograms at 60 min (**B**) and Ca^2+^ imaging (**C**,**D**) of ionophore-treated cells. The images in panels **C** and **D** show pseudocolor representations of cells after 60 min of incubation with A23187 at a concentration of 0.2 µM and 2 µM, respectively. The pictures are representative of three independent experiments, scale bar: 100 µm. Results in the kinetics (**A**) and in the histogram (**B**) are expressed as mean ± SEM. Student’s *t*-test was used to compare the statistical significance. *** *p* < 0.0005 with 109 < *n* < 120.

Finally, to validate the specificity of our previous results, we checked that Ca^2+^ fluxes generated independently of amyloid oligomers could still be detected in SH-SY5Y-AmyP53 cells. To this end, the cells were preloaded with Fluo-4AM and then treated with the Ca^2+^ ionophore A23187. Under these conditions, the transfected SH-SY5Y-AmyP53 cells had, in both cases, dramatic increases in intracellular Ca^2+^ induced by the ionophore. Thus, any Ca^2+^ entry into SH-SY5Y-AmyP53 cells can be easily evidenced by our methodological approaches, thereby excluding any potential artifact due to impaired Ca^2+^ flux measurements in transfected cells (Figure 3).

### 2.3. Neuroprotection Can Be Transferred by Culture Supernatants of AmyP53-Producing SH-SY5Y-AmyP53 Cells

**Figure 4:**
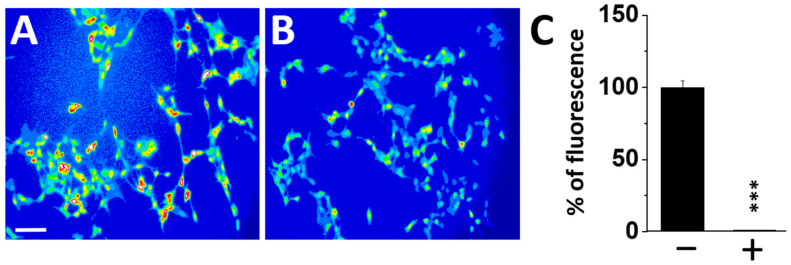
Protection against oligomer formation transferred by cell-free supernatants of SH-SY5Y-AmyP53 cells. Non-transfected SH-SY5Y cells were preloaded with Fluo4 AM, then incubated with Aβ1–42 (220 nM) diluted in culture medium (**A**) or in cell-free cultured supernatant harvested from SH-SY5Y-AmyP53 cells (**B**). The histograms in panel **C** show a quantitative analysis of Ca^2+^ entry in both conditions after 60 min of incubation with Aβ1–42 minus (−) or plus (+) cell-free cultured supernatant harvested from SH-SY5Y-AmyP53 cells. Results in the histogram (**C**) are expressed as mean ± SEM. Student’s *t*-test was used to compare the statistical significance. *** *p* < 0.0005 with 42 < *n* < 65. The pictures are representative of three independent experiments, scale bar: 100 µm.

Altogether, these data suggested that SH-SY5Y-AmyP53 cells are no longer able to generate neurotoxic oligomers because of the chronic secretion of AmyP53 in the culture supernatant, resulting in a paracrine/autocrine mechanism of resistance. If this assumption is correct, it is expected that the culture supernatant of SH-SY5Y-AmyP53 cells could block the formation of neurotoxic oligomers in non-transfected cells, just as exogenously added AmyP53 does. As shown in Figure 4, the supernatant collected from SH-SY5Y-AmyP53 cells efficiently inhibited Ca^2+^ entry in non-transfected cells probed by Aβ1–42.

### 2.4. Protection against Neurite Degeneration

**Figure 5:**
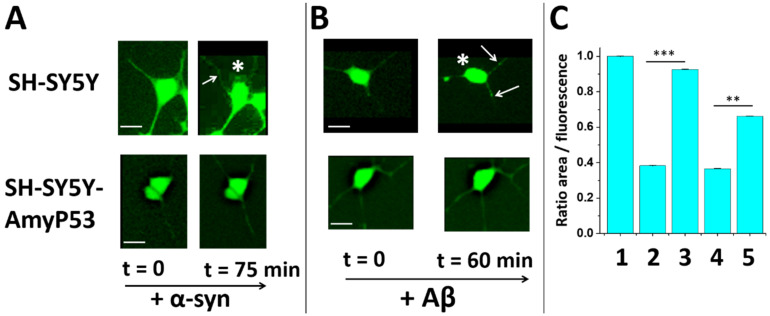
SH-SY5Y-AmyP53 cells are protected from amyloid oligomer-induced neurite degeneration. Neurite degeneration and cell body retraction is a hallmark of the neurotoxicity induced by α-syn (**A**, **upper panels**; scale bar: 20 µm) or Aβ1–42 (**B**, **upper panels**; scale bar: 10 µm) oligomers. These morphological alterations are detected after 60 or 75 min of incubation with amyloid proteins as indicated, which in each case represents the time necessary (i) for the monomers to self-organize into functional Ca^2+^ pores in the plasma membrane of recipient cells, and (ii) for detecting the first signs of neurodegeneration. In contrast, these alterations do not appear in SH-SY5Y-AmyP53 cells after either α-syn (**A**, **lower panels**; scale bar: 10 µm) or Aβ (**B**, **lower panels**; scale bar: 10 µm) incubation. The pictures are representative of three independent experiments. (**C**) Quantitative analysis of cellular surface: (1) control SH-SY5Y cells; (2) Effect of Aβ1–42 (200 nM) on SH-SY5Y cells; (3) Effect of Aβ1–42 (200 nM) on SH-SY5Y-AmyP53 cells; (4) Effect of α-syn (200 nM) on SH-SY5Y cells; (5) Effect of α-syn (200 nM) on SH-SY5Y-AmyP53. Results in the histogram (**C**) are expressed as mean ± SEM. Student’s *t*-test was used to compare the statistical significance. * *p* < 0.05, ** *p* < 0.005; *** *p* < 0.0005 with 45 < *n* < 54. The retraction of the cell bodies induced by the oligomers is indicated by an asterisk and the points of neurite degeneration by the arrows.

One important consequence of oligomer neurotoxicity is a significant reduction in neurites due to the Ca^2+^ overdose [42,43,44]. To check whether SH-SY5Y-AmyP53 cells were also protected from these deleterious events, we performed quantitative measurements of neurite areas in both wild-type and AmyP53-transfected cells probed with either Aβ1–42 or α-syn. Representative micrographs and corresponding histograms are shown in Figure 5. The data showed that both types of oligomers (Aβ1–42 and α-syn) induced a significant contraction of cell bodies and a reduction in neurite areas in control cells. In contrast, such deleterious effects were not observed in SH-SY5Y-AmyP53 cells upon treatment with α-syn or Aβ. Thus, the chronic production of AmyP53 in transfected cells can not only prevent the formation of neurotoxic oligomers, but also suppress all downstream oligomer neurotoxicity events including Ca^2+^ entry and neurite degeneration.

### 2.5. Ganglioside Expression in SH-SY5Y-AmyP53 Cells

**Figure 6:**
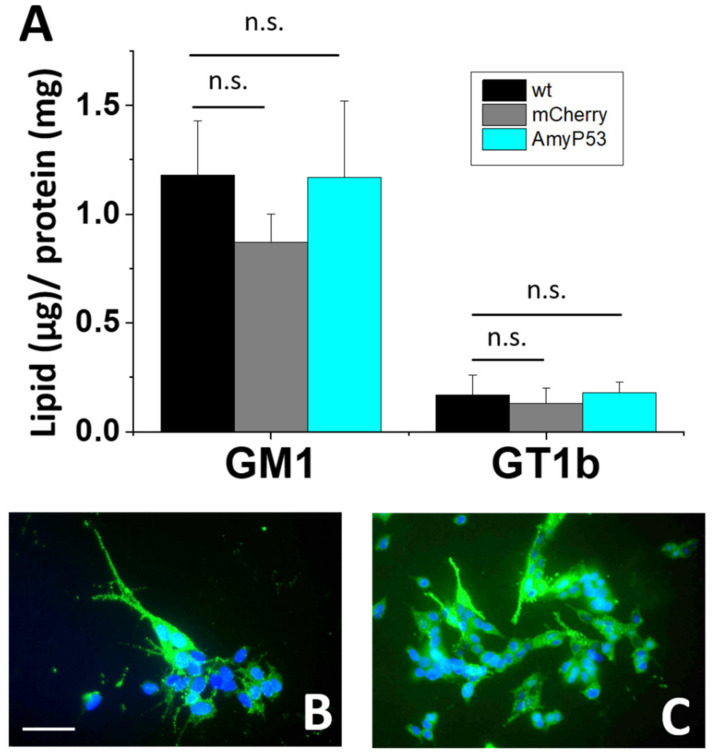
Non-transfected SH-SY5Y and SH-SY5Y-AmyP53 cells have similar ganglioside expression levels. (**A**) HPTLC quantification of gangliosides GM1 and GT1b in non-transfected SH-SY5Y cells (wt) compared with transfected SH-SY5Y-mCherry and SH-SY5Y-AmyP53 cells. Results are expressed as mean ± SEM. Kruskal–Wallis test was used to compare the statistical significance. n.s. > 0.05; *n* = 3. (**B**) Immunofluorescence detection of ganglioside GM1 in non-transfected SH-SY5Y cells. (**C**) Immunofluorescence detection of ganglioside GM1 in SH-SY5Y-AmyP53 cells. The pictures are representative of three independent experiments. Scale bar: 100 µm.

The formation of neurotoxic oligomers in the plasma membrane of brain cells is a coordinated process controlled by raft lipids, especially gangliosides [17,19,30,45]. According to this mechanism, any effect on ganglioside homeostasis could potentially affect oligomer neurotoxicity. Indeed, we previously reported that metabolic inhibition of ganglioside biosynthesis suppressed amyloid pore formation induced by Aβ1–42 and α-syn proteins at the earliest step of neurotoxic oligomer formation [32]. To check this important issue, we studied ganglioside expression in control and AmyP53-transfected cells. As shown in Figure 6, non-transfected SH-SY5Y, SH-SY5Y-mCherry and SH-SY5Y-AmyP53 cells have a similar level of cell surface expression of GM1, a ganglioside previously identified as a key cofactor for neurotoxic oligomers [45]. These data obtained by immunolabeling with anti-GM1 antibodies were confirmed by biochemical analysis of ganglioside (GM1 and GT1b) expression (Figure 6). Thus, the transfection protocol had no effect on the expression of brain gangliosides involved in oligomer formation and neurotoxicity.

### 2.6. Demonstration of the Safety of AmyP53

A critical issue in the development of therapeutic drugs is to demonstrate their safety. The potential toxicity of AmyP53 has been studied in several distinct cellular and animal models. Since inflammation is a current side effect observed upon brain immunotherapies [46,47], we carefully checked the absence of such an undesirable effect for AmyP53. Thus, we analyzed the potential effect of AmyP53 on the levels of inflammatory factors and cytokines that could be produced by cultured neuron and astrocytes. We did not detect any change in the production of the following proinflammatory molecules: IL-1α, IL1-Ra, IL-2, IL-4, IL-5, IL-6, IL-7, IL-8, IL-9, IL-10, IL-12, IL-13, IL-15; IL-17, eotaxin, PDGF, FGF, GM-CSF, GM-CSF, IFNγ, IP10, MCP1, MIP1 α, MIP1β, RANTES, TNFα and vEGF (Figure S4).

Then, determination of the maximal tolerated dose (MTD) and dose ranging finding (DRF) studies of AmyP53 were assessed in rats treated daily by either intranasally or intravenous administration (Tables S1 and S2 and Figure S5). No toxicity in these MTD and DRF studies has been observed, even at the maximal dose of 5 mg/kg body weight (Table S1, intranasal administration) and 80 mg/kg body weight (Table S2, intravenous administration). In particular, AmyP53 did not affect body weight (Tables S1 and Table S2) or food consumption (Figure S5).

Moreover, careful examination of the injection zones in animals treated at the highest doses did not reveal any sign of inflammation (Figure S6).

A dose ranging finding (DRF) study conducted in rats confirmed the total lack of toxicity of AmyP53 even at the highest doses tested, i.e., 5 mg/kg body weight for intranasal administration. A summary of DRF data is presented in Table S3 (hematological markers), Table S4 (blood chemistry parameters) and Table S5 (enzymatic parameters). Overall, these data indicated that AmyP53 has a good safety profile in rodents, whatever the route of administration (intranasal or intravenous).

## 3. Discussion and Conclusions

### 3.1. Discussion

AmyP53 is a therapeutic peptide targeting brain gangliosides that has been rationally designed for jamming the process of amyloid pore formation [30]. AmyP53 prevents the generation of these neurotoxic oligomers at the earliest step [19,31,32], which is the binding of the amyloid protein (α-syn or Aβ) to ganglioside-enriched lipid rafts at the surface of brain cells [28]. The essential role of gangliosides in neurological diseases, anticipated a decade ago [17], has now received consensual recognition [45,48,49,50]. Thus, targeting brain membrane gangliosides is considered as a golden opportunity to design novel therapeutic strategies through innovative approaches [9,28,51,52,53,54]. AmyP53 is the first molecule that tackles gangliosides, prevents the formation of neurotoxic oligomers and is active against two amyloid proteins (α-syn and Aβ), including mutant forms that are responsible for inherited Parkinson and Alzheimer diseases [28,32].

AmyP53 has been specifically designed to reach the exact pool of brain gangliosides recognized by amyloid proteins during the process of oligomer formation. This pool consists of dimers of gangliosides that interact chiefly with their membrane-embedded ceramide parts, resulting in the formation of a large chalice-like landing area (Figure 2), recognized by the typical turn-conformers of amyloid proteins [26,29,30]. AmyP53 mimics the active turn conformation of α-syn and Aβ, but displays a much higher avidity and a better molecular fit for gangliosides than each of the amyloid proteins from which it is derived [30]. This unique property, intentionally created by computational design, explains why AmyP53 works at such low concentrations, with an EC50 of 30 nM in competition with 220 nM of amyloid protein in our amyloid pore assay (Figure S2).

In this study, we demonstrate the successful transfection of a coding region mixing the signal sequence of human serum albumin and AmyP53 in a retroviral vector. We created a stable cell line (SH-SY5Y-AmyP53) which constitutively secretes the AmyP53 peptide in the culture supernatant. These cells are totally resistant to the neurotoxicity of amyloid oligomers, including those formed by α-syn and Aβ, consistent with the amounts of AmyP53 detected in the culture supernatants of transfected cells which largely exceed the minimal therapeutic dose.

As expected, the protection against oligomers could be transferred to non-transfected cells by culture supernatants harvested from SH-SY5Y-AmyP53. This result suggests that only a few brain cells need to be transfected to prevent oligomer formation in vicinal cells. The level of protection was assessed by the blockade of Ca^2+^ entry induced by amyloid oligomers, and all subsequent neurotic events that result in neurodegeneration (neurite degeneration and cell body contraction).

The neuroprotection achieved in SH-SY5Y-AmyP53 cells was totally dependent upon the transfection with the AmyP53 coding sequence, as both non-transfected cells and control SH-SY5Y-mCherry cells are highly sensitive to oligomer formation. We also demonstrated that the transfection did not alter ganglioside levels. Thus, the resistance of SH-SY5Y cells to oligomer formation could not be attributed to impaired ganglioside expression but to competitive inhibition of ganglioside-dependent membrane binding of amyloid by AmyP53.

To the best of our knowledge, this is the first time that a gene therapy specifically targets the common pathway of neurotoxic oligomer formation. For this reason, our study opens a new route for a unique strategy for AD and PD.

The potential toxicity of AmyP53 has been carefully studied in several in vitro and in vivo models. First, the peptide did not trigger any inflammatory reaction or chemokine secretion as assessed by quantitative measurements of 27 factors released by cultured neurons and astrocytes. Second, no tissue inflammation was observed at the site of intravenous and intranasal injections of AmyP53 in rats (Figure S6). Moreover, the animals that received up to 5 mg/kg of body weight (intranasal administration) and 80 mg/kg (intravenous injection) did not show any sign of toxicity as assessed by physiological, histological and biochemical markers. These data confirmed that AmyP53 does not interact with gangliosides involved in critical functions, and thus strengthens the notion that the peptide targets the pool of gangliosides recognized by extracellular amyloid proteins. The lack of toxicity of AmyP53 is also consistent with its design, as it combines the ganglioside binding domains of α-syn and Aβ. These domains belong to the non-toxic regions of the proteins, which are distinct from the regions that control the oligomerization process [27,28,30,37]. In this respect, it is interesting to note that both Aβ and α-syn display intrinsically disordered regions that undergo structuration upon binding to lipid rafts [9]. Indeed, the common ganglioside binding domains of these proteins consists in a surface accessible loop that is shaped by selected gangliosides acting as lipid chaperones [9,17,19,31,54]. By targeting this specific pool of gangliosides, AmyP53 may block this key chaperone activity at the earliest step, i.e., before the binding of amyloid proteins to brain cells.

### 3.2. Conclusions

In conclusion, the goal of this study was to evaluate the possibility of a gene therapy that could efficiently prevent the formation of neurotoxic oligomers that are considered to be the initial root cause of neurodegenerative diseases, including AD and PD. As a matter of fact, therapeutic peptides have several advantages compared with small molecules and antibodies [19]: they are less toxic, more specific and can be administered by several routes, including intravenous, intranasal, oral and subcutaneous [19,34]. In the case of AmyP53, both the intravenous and intranasal pathways have been validated by the MTD and DRF studies presented in this study. The data obtained by genetic transfer of the AmyP53 coding sequence in cultured brain cells further extends these possibilities to a gene therapy aimed at controlling the formation of neurotoxic oligomers by targeting brain gangliosides. This proof of concept is a decisive step in the development of AmyP53 as a therapeutic solution for neurological disorders including AD and PD. Beyond the demonstrations of (i) a good safety profile in rodents, and (ii) a therapeutic efficiency at nanomolar concentrations against a broad range of neurotoxic oligomers (thus potentially usable for AD and PD treatment), these data indicate that AmyP53 has now two de-risked routes of administration (intranasal and intravenous) and two conceivable formulations (soluble preparation or coded in a viral vector). The recent breakthrough in mRNA vaccine technologies [55,56] raises the interesting possibility of using therapeutic RNAs as possible carriers of the AmyP53 coding sequence in selected brain areas. In the meantime, AmyP53 will be evaluated in phase I clinical trials, preferentially in a nasal spray formulation for the best possible comfort of patients, their families and health care workers.

## 4. Materials and Methods

### 4.1. Products

SH-SY5Y and CTX-TN2A (see supplementary materials) cells were purchased from ATCC (Manassas, VA, USA). DMEM/F12, HBSS, glutamine and penicillin/streptomycin were furnished by Gibco (Amarillo, TX, USA). Fluo-4AM and secondary antibody were purchased from Invitrogen (Waltham, MA, USA). The anti-ganglioside GM1 antibody was purchased from Matreya (State College, PA, USA). The full-length proteins α-synuclein1–140 and Aβ1–42 were from rPeptide (Watkinsville, GA, USA). These proteins were dissolved in 1% NH4OH at a concentration of 1 mM and frozen at −20 °C in working aliquots. The chimeric AmyP53 peptide was obtained from Schafer-N (København, Denmark). All peptides and proteins have a purity >95% as assessed by HPLC. The chimeric peptide used in this study has been patented under the number PCT/EP2015/054968: “A chimeric peptide that interacts with cell membrane gangliosides”.

### 4.2. Vector Construction and Lentiviral Transduction

The HIV-derived lentiviral vector pRRL/iRFP-IRES-mCherry, equipped with the internal ribosomal entry sequence (IRES) to allow for expression of a single biscistronic mRNA encoding both iRFP and mCherry has been described previously [57]. In the present study, the mCherry sequence was swapped with that of the chimeric AmyP53 peptide, thus driving the expression of the latter with the near-infrared (NIR) fluorescent protein iRFP. The resulting plasmid was named pRRL/iRFP-IRES-a-Syn. The highly penetrative near-infrared fluorescence [58] would allow for continuous monitoring of the peptide-secreting cells, e.g., when inoculated to an established preclinical animal model. To this end, the forward and reverse oligonucleotides (Eurogentec, Angers, France) encompassing the human serum albumin signal peptide sequence (Genbank#AY960291.1) followed by that of the chimeric AmyP53 peptide [30], were annealed and cloned into pRRL/iRFP-IRES-mCherry cut with *EcoR*V and *Sal*I to remove the mCherry DNA. Lentiviral particles preparation and infection of cells with viral particles were performed as described previously [59].

### 4.3. Cell Culture

CTX-TN2A, non-transfected SH-SY5Y and SH-SY5Y transfected with AmyP53 (SH-SY5Y-AmyP53) were cultured in Dulbecco’s Modified Eagle Medium: Nutrient Mixture F12 (DMEM/F12) supplemented with 10% fetal calf serum, glutamine (2 mM) and penicillin (50 U/mL)/streptomycin (50 μg/mL) and maintained at 37 °C with 5% CO_2_.

### 4.4. Cell Surface Labeling and Dosing of AmyP53

Custom rabbit polyclonal antibodies against AmyP53 (affinity-purified, titer > 1:50,000) were purchased from Clinisciences (Nanterre, France). For immunological staining, SH-SY5Y cells were fixed with 4% formaldehyde, rinsed and incubated with anti-AmyP53 (1:500) for 2 h, rinsed and subsequently treated with goat anti-rabbit Alexa Fluor 488 (1:400) for 1 h [32]. ELISA dosing of AmyP53 was performed using a standard procedure used for short synthetic peptides [60].

### 4.5. Calcium Assay and Neurite Degeneration

Non-transfected SH-SY5Y and SH-SY5Y-AmyP53 cells were loaded with 5 μM Fluo-4AM for 30 min in the dark, washed three times with HBSS and incubated 30 min at 37 °C. Calcium fluxes were estimated by measuring the variation in cell fluorescence intensity after amyloid protein injection (220 nM) into the recording chamber directly above an upright microscope objective (BX51W Olympus, Tokyo, Japan) equipped with an illuminator system MT20 module [32]. Fluorescence emission at 525 nm was imaged by a digital camera CDD (Hamanatsu ORCA-ER, Hamamatsu, Japan) after fluorescence excitation at 490 nm. Time-lapse images (1 frame/10 s) were collected using the CellR Software (Olympus). Fluorescence intensity was measured from region of interest (ROI) centered on individual cells. Signals were expressed as fluorescence after treatment (F_t_) divided by the fluorescence before treatment (F_t0_) and multiplied by 100. The results were averaged and the fluorescence of control untreated cells was subtracted of each value. All experiments were performed at 30 °C during 60–75 min.

At the end of the calcium assay, fluorescent images were taken. In each condition, the morphology of cells was traced, the area and the fluorescent intensity were determined using the ImageJ software. Then, the ratio area/fluorescence was determined for each cell and averaged for each condition.

### 4.6. Immunological Detection and Quantification of Gangliosides

For immunological staining, the non-transfected SH-SY5Y and the SH-SY5Y-AmyP53 cells were incubated with anti-ganglioside GM1 primary antibody (1:500) for 2 h, rinsed and subsequently treated with goat anti-rabbit Alexa Fluor 488 (1:400) for 1 h [32]. Gangliosides were extracted from the different cell lines and recovered from the upper phase of a Folch partition, analyzed by high performance thin layer chromatography (HPTLC) and quantitated with a Gel Doc™ XR + Molecular Imager using the Image Lab™ software as previously described [32].

### 4.7. Quantification of Chemokines and Proinflammatory Factors

The non-transfected SH-SY5Y and the CTX-TN2A cells were plated on 96-well plates and treated with 10 µM of AmyP53 for 24 h at 37 °C. After 24 h of incubation, the supernatants were collected and chemokines and proinflammatory factors were quantified by Bioplex Multiplex Immunoassay System (Bio-Rad, Hercules, CA, USA) according to manufacturer’s instructions.

### 4.8. In Silico Studies

Molecular dynamics simulations of ganglioside–peptide interactions have been performed with the Hyperchem and Molegro programs as already described [61,62].

### 4.9. Toxicology Studies

All animal studies have been performed by EtapLab (http://www.etap-lab.com/, accessed on 1 August 2019), Vandoeuvre-les-Nancy, France. The Toxicology Department of this CRO has obtained the ISO 9001:v2008 certification for “Consulting, advising expertise in toxicology, studies in toxicology performed or conducted by the Toxicology Department”. This company is in full compliance with European guidelines for animal testing, ISO 9001 standards and 21 CFR 58 GLP Regulations on data management and traceability and are in accordance with the European directive concerning animal experimentation (2010/63/EU, Government authorization No. 54-85/2012). The experiments have been approved by the CELMEA ethics committee (AMYPORE/P4-T-0919/AmyP53/DRF-IN/v1 and AMYPORE/P3-T-0719/AmyP53/IV-IN/v1) and the study has been carried out in compliance with the ARRIVE guidelines. Wistar rats (8 weeks) had access to food and water ad libitum and were housed under a 12-h light/dark cycle at 22–24 °C. For animal studies, endotoxin-free and solvent-free AmyP53 has been synthesized under GMP-like conditions at a purity >98% by Proteogenix, Schiltigheim, France.

### 4.10. Statistical Analysis

All data were expressed as mean ± S.E.M. and the statistical significance was tested using Student’s *t*-test, one way ANOVA or Kruskal–Wallis test (non-parametric test).

## Supplementary Materials

The following are available online at https://www.mdpi.com/article/10.3390/ijms222111550/s1, Figure S1: Dosing of AmyP53 in cell culture supernatants by ELISA, Figure S2: Dose-dependent effect of AmyP53 on amyloid pore formation, Figure S3: Amyloid pore formation induced by α-syn in SH-SY5Y-mCherry cells, Figure S4: AmyP53 does not affect the production of chemokines and proinflammatory factors in cultured neurons and astrocytes, Figure S5: Food consumption of female rats (g/kg BW) of the 2 experimental groups injected intravenously (IV) and intranasally (IN) with increasing doses if aqueous solutions of AmyP53, Figure S6: Safety of AmyP53 intranasal (A) and intravenous (B, C) administration of AmyP53 in rats, Table S1: DRF study of body weights of rats of the 2 experimental groups injected intranasally with doses of aqueous solutions of AmyP53 of 0.2, 1.0 and 5.0 mg/kg body weight (BW) every two days between D1 and D5, Table S2: MTD study of mean body weights of rats of the 2 experimental groups injected intravenously and intranasally with increasing doses of aqueous solutions of AmyP53 ranging from 0.128 to 80.0 mg/kg BW for the intravenous route and from 0.0064 to 4.0 mg/kg BW for the intranasal route every two days between D6 and D10, Table S3: Comparison of hematological parameters of female rats of the 4 experimental groups injected intranasally with doses of aqueous solutions of AmyP53 of 0.2, 1.0 and 5.0 mg/kg BW every two days between D1 and D5, at D7, Table S4: Comparison of blood chemistry parameters of female rats of the 4 experimental groups injected intranasally with doses of aqueous solutions of AmyP53 of 0.2, 1.0 and 5.0 mg/kg BW every two days between D1 and D5, at D7, Table S5: Comparison of enzymatic parameters of female rats of the 4 experimental groups injected intranasally with doses of aqueous solutions of AmyP53 of 0.2, 1.0 and 5.0 mg/kg BW every two days between D1 and D5, at D7.
