# Supplementary material for: Gene Therapy Strategy for Alzheimer’s and Parkinson’s Diseases Aimed at Preventing the Formation of Neurotoxic Oligomers in SH-SY5Y Cells
Source: Int J Mol Sci. 2021 Oct 26;22(21):11550. doi: 10.3390/ijms222111550 (PMC8583875; doi:10.3390/ijms222111550)
Supplement: Supplementary file 1 [file ijms-22-11550-s001.zip › ijms-1419579-supplementary.pdf]

Supplementary information

Gene therapy strategy for Alzheimer's and Parkinson's diseases aimed at preventing the formation of neurotoxic oligomers

Assou El-Battari[1], Léa Rodriguez[2], Henri Chahinian[1], Olivier Delézay[3], Jacques Fantini[1], Nouara Yahi[1] and Coralie Di Scala[4*]

1- INSERM UMR_S 1072, 13015 Marseille, France; Aix-Marseille Université, 13015 Marseille, France.
2- CUO-Recherche, Département d'ophtalmologie, Faculté de médecine, Université Laval and Centre de recherche du CHU de Québec-Université Laval, Québec, Canada.
3- SAINBIOSE INSERM U1059 Campus Santé Innovations, Faculté de Médecine ST Priest en Jarez, France.
4- Neuroscience Center-HiLIFE, University of Helsinki, 00014, Helsinki, Finland.

*Corresponding author: Coralie Di Scala, e-mail: coralie.discala@helsinki.fi

1. Figure S1: Dosing of Amyp53 in cell culture supernatants by ELISA.

2. Figure S2: Dose-dependent effect of AmyP53 on amyloid pore formation.

3. Figure S3: Amyloid pore formation induced by α-syn in SH-SY5Y-mCherry cells.

4. Figure S4: AmyP53 does not affect the production of chemokines and proinflammatory factors in cultured neurons and astrocytes.

5. Results of a maximal tolerated dose (MTD) and dose ranging finding (DRF) studies of AmyP53 in rats.

- Table S1. DRF study of body weights of female rats of the 4 experimental groups injected intranasally with doses of aqueous solutions of AmyP53 of 0.2, 1.0 and 5.0 mg/kg body weight (BW) every two days between D1 and D5.

- Table S2. MTD study of mean body weights of rats of the 2 experimental groups injected intravenously and intranasally with increasing doses of aqueous solutions of

AmyP53 ranging from 0.128 to 80.0 mg/kg BW for the intravenous route and from 0.0064 to 4.0 mg/kg BW for the intranasal route every two days between D6 and D10

- Figure S5. Food consumption of female rats (g/kg BW) of the 2 experimental groups injected intravenously (IV) and intranasally (IN) with increasing doses of aqueous solutions of AmyP53.

- Conclusion of the MTD study.

6. Figure S6: Safety of AmyP53 intranasal (A) and intravenous (B, C) administration of AmyP53 in rats.

7. Summary of dose ranging finding (DRF) data: hematological, blood chemistry, and enzymatic parameters.

- Table S3. Comparison of hematological parameters of female rats of the 4 experimental groups injected intranasally with doses of aqueous solutions of AmyP53 of 0.2, 1.0 and 5.0 mg/kg BW every two days between D1 and D5, at D7.

- Table S4. Comparison of blood chemistry parameters of female rats of the 4 experimental groups injected intranasally with doses of aqueous solutions of AmyP53 of 0.2, 1.0 and 5.0 mg/kg BW every two days between D1 and D5, at D7.

- Table S5. Comparison of enzymatic parameters of female rats of the 4 experimental groups injected intranasally with doses of aqueous solutions of AmyP53 of 0.2, 1.0 and 5.0 mg/kg BW every two days between D1 and D5, at D7.

- Conclusion of the DRF study.

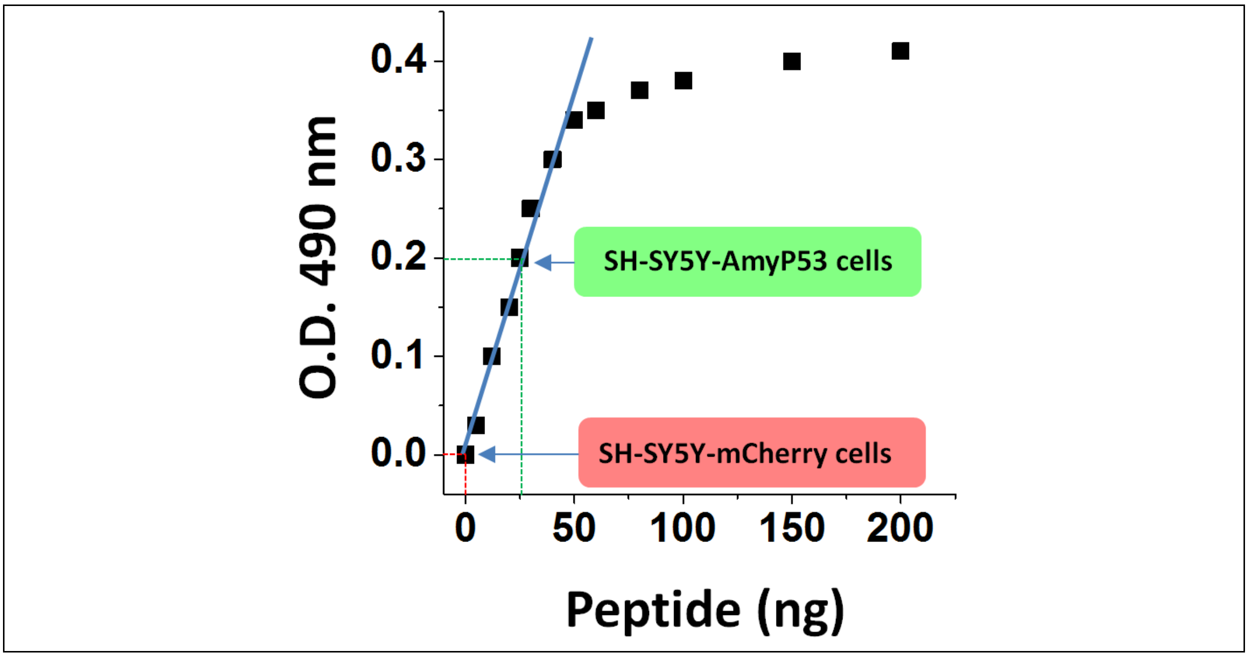

<u>Figure S1</u>: Dosing of AmyP53 in cell culture supernatants by ELISA.

ELISA plates were coated with synthetic AmyP53 and incubated with rabbit anti-AmyP53 antibodies. After rinsing the plates were incubated with goat peroxydase-coupled goat anti-rabbit antibodies, rinsed and revealed with SIGMAFAST^{TM} OPD (o-Phenylenediamine dihydrochloride) substrate. The reaction was stopped with H_2SO_4 and the absorbance was read at 490 nm. Several determinations of the amount of AmyP53 in serially diluted cell culture supernatants were performed. A typical example illustrates here the detection of immunoreactive AmyP53 in culture supernatants harvested from SH-SY5Y-AmyP53 cells and the lack of detection of the peptide in the case of control SH-SY5Y-mCherry cells.

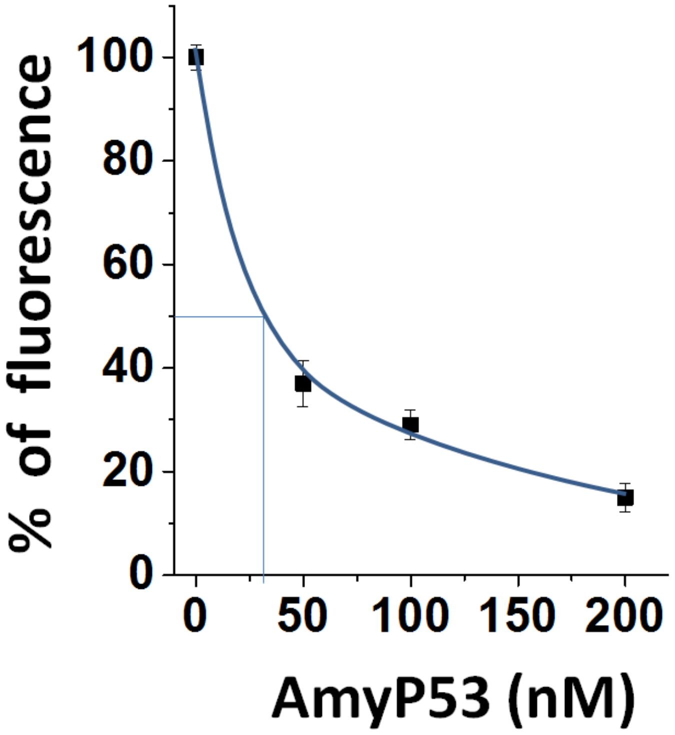

Figure S2: Dose-dependent effect of AmyP53 on amyloid pore formation.

Non-transfected SH-SY5Y cells were preloaded with Fluo-4AM, then incubated with Aβ1-42 (220 nM) in absence or presence of various concentrations of AmyP53 in competition. The data show the amounts of intracellular Ca^{2+} after 60 minutes of incubation. The EC50 of AmyP53 was estimated to 30 nM.

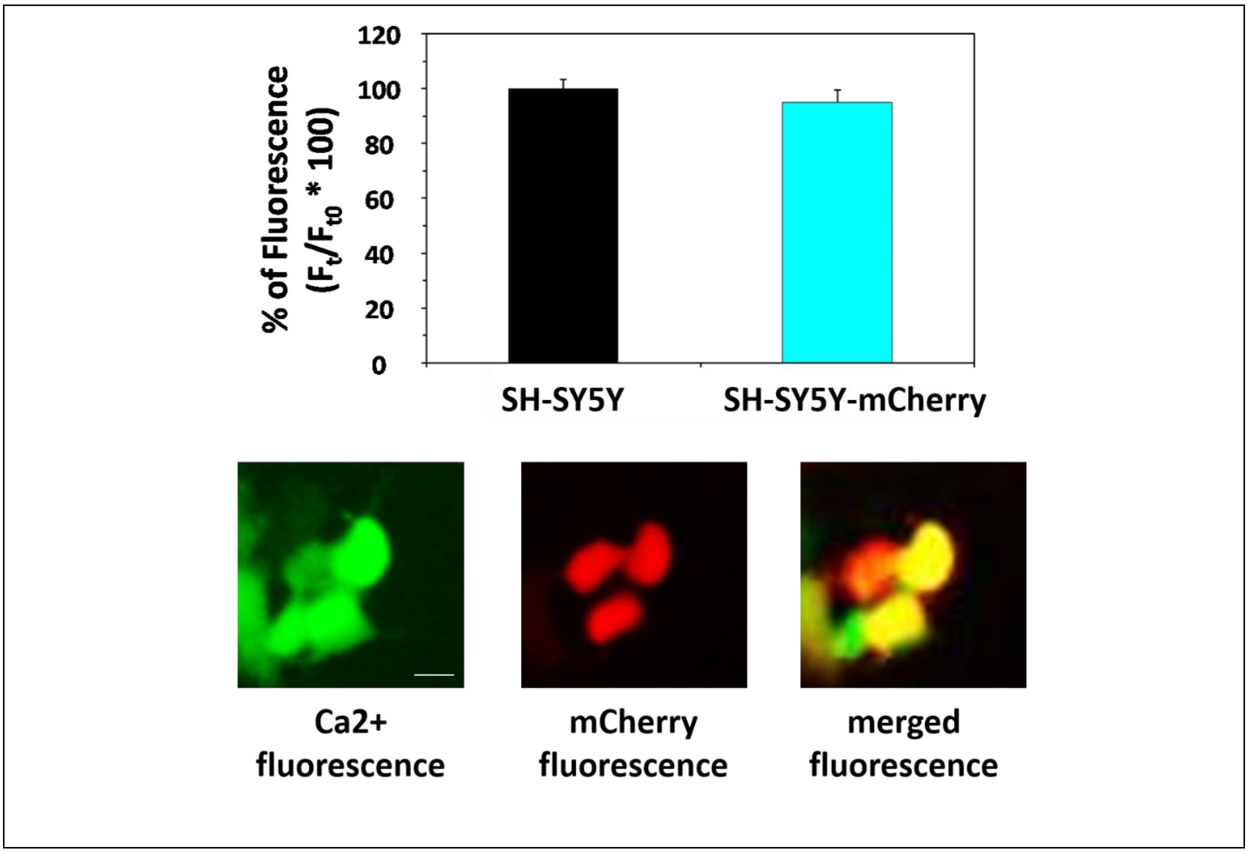

Figure S3: Amyloid pore formation induced by α-syn in SH-SY5Y-mCherry cells. SH-SY5Y-mCherry cells were used as control of transfection. These cells were preloaded with Fluo-4AM, and then incubated with α-syn (200 nM). The histograms in the upper panel show that the amounts of intracellular Ca^{2+} in non-transfected SH-SY5Y (n = 100) and in SH-SY5Y-mCherry cells (n = 102) after 75 minutes of incubation with α-syn are remarkably similar and are expressed as mean ± SEM.. The fluorescence micrographs in the lower panel indicate that the cells which respond to α-syn are indeed transfected cells, since these cells display the typical mCherry fluorescence. Scale bar: 1 µm.

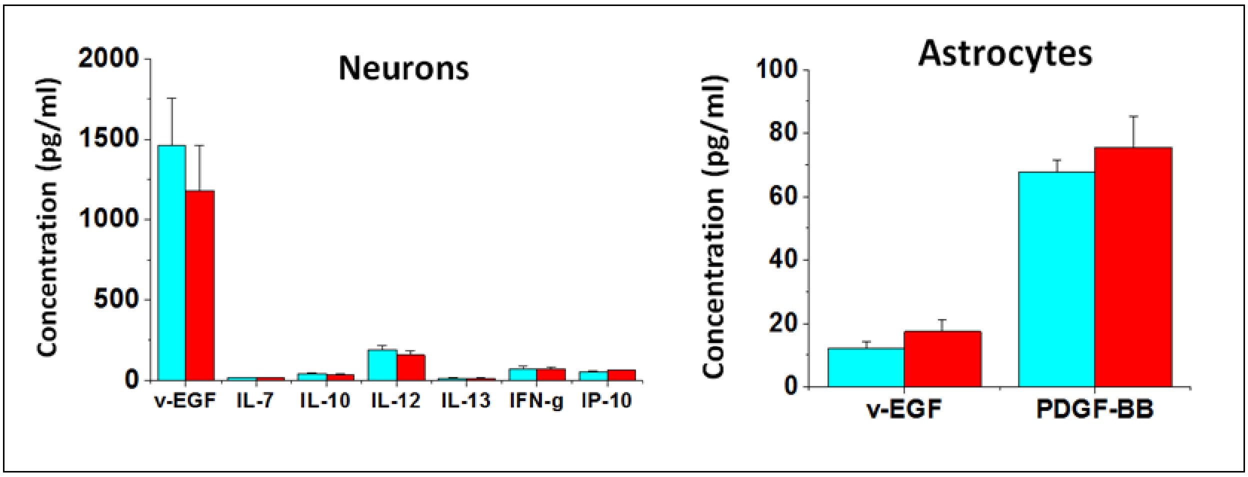

<u>Figure S4</u>: AmyP53 does not affect the production of chemokines and proinflammatory factors in cultured neurons and astrocytes. The data show the concentration of each detected factor in culture supernatants from neurons (SH-SY5Y) and astrocytes (CTX) harvested in either the absence or presence of AmyP53 (10 µM) during 24hr (respectively blue or red histograms). Apart from these 8 factors, we did not detect any of the following 19 chemokines in either the absence or presence of AmyP53 (10 µM, 24-hr): IL-1β, IL-1ra, IL-2, IL-4, IL-5, IL-6, IL-8, IL-9, IL-15, IL-17, eotaxin, FGF, G-CSF, GM-CSF, MCP-1, MIP1α, MIP-1α, RANTES, TNF-α. The concentrations of these factors were quantified using a Bioplex Multiplex Immunoassay System (Bio-Rad).

Results of a maximal tolerated dose (MTD) and dose ranging finding (DRF) studies of AmyP53 in rats.

Treatment	D1	D2	D3	D4	D5	D6	D7
Vehicle	169.78 ± 5.99	173.98 ± 5.62	179.28 ± 5.72	182.51 ± 5.89	184.79 ± 5.25	188.55 ± 5.72	192.46 ± 5.44
AmyP53 0.2 mg/kg	166.52 ± 13.59	171.28 ± 13.70	177.52 ± 14.39	180.80 ± 16.15	182.41 ± 18.02	186.05 ± 17.66	189.69 ± 15.15
AmyP53 1.0 mg/kg	167.36 ± 1.53	172.48 ± 1.89	177.64 ± 2.93	182.33 ± 3.28	182.27 ± 2.60	185.49 ± 3.27	191.03 ± 3.13
AmyP53 5.0 mg/kg	168.41 ± 3.46	170.47 ± 4.43	173.11 ± 5.10	177.96 ± 5.10	179.71 ± 4.16	182.08 ± 5.14	185.70 ± 5.77
Kruskal-Wallis Significance	$H_{(3df)} = 0.74$ *NS*	$H_{(3df)} = 0.74$ *NS*	$H_{(3df)} = 0.74$ *NS*	$H_{(3df)} = 1.15$ *NS*	$H_{(3df)} = 0.74$ *NS*	$H_{(3df)} = 0.90$ *NS*	$H_{(3df)} = 0.90$ *NS*

Table S1: DRF study of body weights of female rats of the 4 experimental groups injected intranasally with doses of aqueous solutions of AmyP53 of 0.2, 1.0 and 5.0 mg/kg BW every two days between D1 and D5 (n=3) (Mean ± SEM).

Group	D_6	D_7	D_8	D_9	D_{10}
Intravenous	180.4 ± 6.2	185.1 ± 6.0	188.0 ± 5.4	191.2 ± 4.5	193.0 ± 4.4
Intranasal	177.4 ± 6.4	180.0 ± 5.9	184.0 ± 5.7	187.5 ± 7.2	188.7 ± 6.6
K-W U-test Significance	U=6.0 *P=0.56*	U=6.0 *P=0.56*	U=6.0 *P=0.56*	U=7.0 *P=0.77*	U=6.0 *P=0.56*

Table S2: MTD study of mean body weights of rats of the 2 experimental groups injected intravenously and intranasally with increasing doses of aqueous solutions of AmyP53 ranging from 0.128 to 80.0 mg/kg BW for the intravenous route and from 0.0064 to 4.0 mg/kg BW for the intranasal route every two days between D6 and D10 (n=4) (Mean ± SEM).

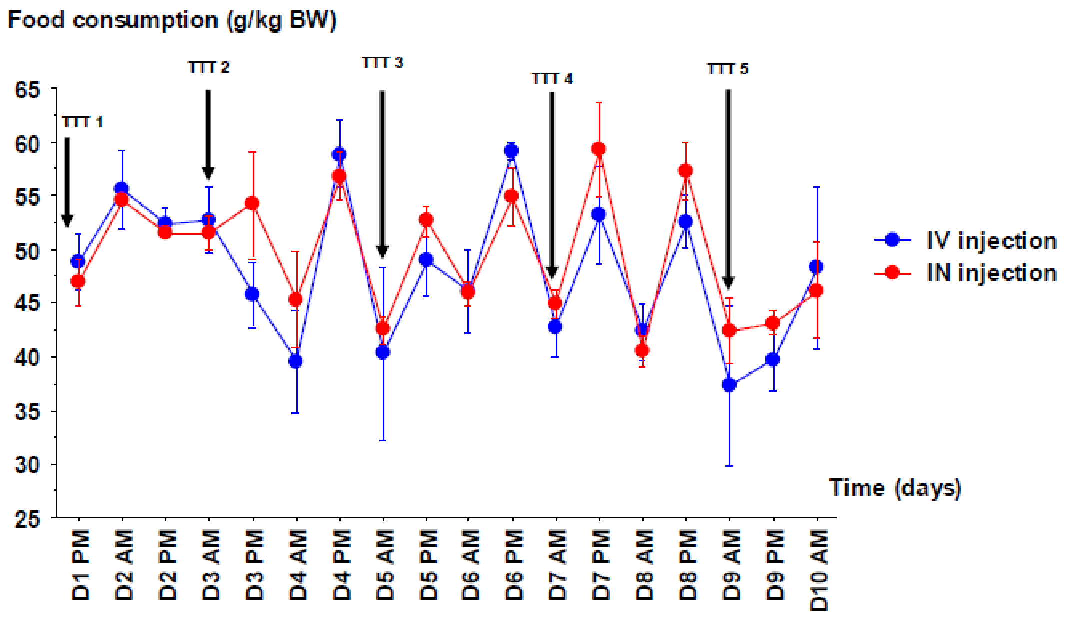

Figure S5: Food consumption of female rats (g/kg BW) of the 2 experimental groups injected intravenously (IV) and intranasally (IN) with increasing doses of aqueous solutions of AmyP53 ranging from 0.128 to 80.0 mg/kg BW for the intravenous route and from 0.0064 to 4.0 mg/kg BW for the intranasal route every two days between D1 and D9 (n=2) (Mean ± SEM).

Conclusion of the MTD study: No mortality, direct or induced, no signs of dysfunction, no body weight loss, no behavioral changes, no modification of food consumption and no abnormality of all the organs of the thoracic and abdominal cavities at autopsy were observed after repeated intravenous and intranasal injections of increasing doses of aqueous solutions of AmyP53 ranging from 0.128 to 80.0 mg/kg BW for the intravenous route and 0.0064 to 4.0 mg/kg BW for the intranasal route in female Wistar rats. The maximal tolerated dose (MTD) of AmyP53 in female Wistar rats is greater than 80.0 mg/kg BW for the intravenous route and greater than 4.0 mg/kg BW for the intranasal route.

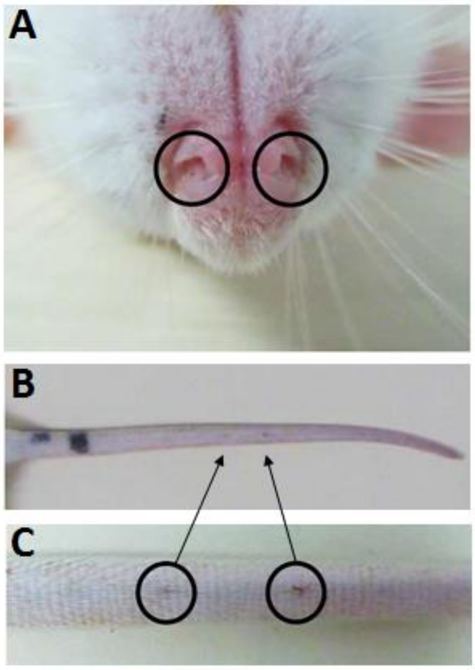

<u>Figure S6</u>: Safety of AmyP53 intranasal (A) and intravenous (B, C) administration of AmyP53 in rats. A- Nostrils seen from below. **B, C-** Intravenous injection sites in the left tail vein. These photographs were taken at the end of the MTD study after the last administration of AmyP53 (highest dose is 4 mg/kg and 80 mg/kg respectively for intranasal and intravenous injections).

Summary of dose ranging finding (DRF) data: hematological, blood chemistry, and enzymatic parameters.

Treatment	RBC (/mm^3)	HG (g/dl)	Hematocrit (%)	MGV (fL)	MCH (pg)	MCHC (g/dl)	WBC (/mm^3)
Vehicle	7.25x10^6 ± 2.91x10^5	13.97 ± 0.07	43.67 ± 0.15	60.00 ± 0.00	19.23 ± 0.12	32.00 ± 0.17	2663.3 ± 337.9
AmyP53 0.2 mg/kg	7.090x10^{6*} ± 2.40x10^5	13.55* ± 0.25	42.95* ± 0.85	60.50* ± 0.50	19.10* ± 0.30	31.55* ± 0.05	2285.0* ± 95.0
AmyP53 1.0 mg/kg	7.37x10^6 ± 1.20x10^5	14.17 ± 0.23	44.87 ± 0.72	61.00 ± 0.58	19.20 ± 0.32	31.57 ± 0.18	3126.7 ± 529.2
AmyP53 5.0 mg/kg	7.30x10^6 ± 1.93x10^5	14.13 ± 0.37	44.50 ± 1.33	61.00 ± 0.00	19.33 ± 0.09	31.77 ± 0.26	2600.0 ± 550.1
Kruskal-Wallis Significance	$H_{(3df)}$ = 1.76 *NS*	$H_{(3df)}$ = 0.31 *NS*	$H_{(3df)}$ = 3.99 *NS*	$H_{(3df)}$ = 4.08 *NS*	$H_{(3df)}$ = 0.23 *NS*	$H_{(3df)}$ = 3.26 *NS*	$H_{(3df)}$ = 1.22 *NS*

RBC = red blood cells; HG = hemoglobin; MGV = mean globular volume; MCH = mean corpuscular hemoglobin; MCHC = mean corpuscular hemoglobin concentration; WBC = white blood cells.

Table S3. Comparison of hematological parameters of female rats of the 4 experimental groups injected intranasally with doses of aqueous solutions of AmyP53 of 0.2, 1.0 and 5.0 mg/kg BW every two days between D1 and D5, at D7 (n=3) (Mean ± SEM). Similar results were obtained with male rats.

Treatment	Glucose (g/l)	Total prot (g/l)	Albumin (g/l)	Sodium (mEq/l)	Potassium (mEq/l)	Chloride (mEq/l)
Vehicle	1.62 ± 0.03	57.40 ± 0.75	43.33 ± 0.33	143.33 ± 0.88	6.80 ± 0.10	96.33 ± 0.88
AmyP53 0.2 mg/kg	1.64 ± 0.20	57.30 ± 1.15	42.33 ± 1.20	144.67 ± 0.67	6.57 ± 0.48	99.33 ± 0.88
AmyP53 1.0 mg/kg	1.83 ± 0.07	56.13 ± 1.23	43.00 ± 1.53	144.33 ± 1.33	5.60 ± 0.25	98.67 ± 0.67
AmyP53 5.0 mg/kg	1.89 ± 0.04	55.37 ± 0.78	43.33 ± 0.88	143.67 ± 0.33	5.57 ± 0.32	98.33 ± 1.33
Kruskal-Wallis Significance	$H_{(3df)}$ = 3.92 *NS*	$H_{(3df)}$ = 3.04 *NS*	$H_{(3df)}$ = 0.55 *NS*	$H_{(3df)}$ = 1.76 *NS*	$H_{(3df)}$ = 5.42 *NS*	$H_{(3df)}$ = 4.35 *NS*

Total Prot = total proteins.

Table S4. Comparison of blood chemistry parameters of female rats of the 4 experimental groups injected intranasally with doses of aqueous solutions of AmyP53 of 0.2, 1.0 and 5.0 mg/kg BW every two days between D1 and D5, at D7 (n=3) (Mean ± SEM). Similar results were obtained with male rats.

Treatment	ASAT (IU/l)	ALAT (IU/l)	γGT (IU/l)	Alkaline P (IU/l)	Total Chol. (g/l)	Urea (g/l)	Creatinin (mg/ml)
Vehicle	344.33 ± 84.72	73.00 ± 18.58	3.00 ± 0.00	174.33 ± 1.20	0.84 ± 0.08	0.41 ± 0.02	2.57 ± 0.03
AmyP53 0.2 mg/kg	257.67 ± 26.03	72.67 ± 11.05	3.00 ± 0.00	169.33 ± 3.38	0.80 ± 0.12	0.34 ± 0.03	2.33 ± 0.12
AmyP53 1.0 mg/kg	197.00 ± 18.03	55.00 ± 2.52	3.00 ± 0.00	165.00 ± 37.00	0.67 ± 0.08	0.39 ± 0.02	2.60 ± 0.00
AmyP53 5.0 mg/kg	165.00 ± 43.68	44.33 ± 13.09	3.00 ± 0.00	165.00 ± 36.59	0.71 ± 0.01	0.34 ± 0.05	2.63 ± 0.12
Kruskal-Wallis Significance	$H_{(3df)}$ = 3.92 *NS*	$H_{(3df)}$ = 3.09 *NS*	$H_{(3df)}$ = 0.00 *NS*	$H_{(3df)}$ = 1.13 *NS*	$H_{(3df)}$ = 2.78 *NS*	$H_{(3df)}$ = 2.69 *NS*	$H_{(3df)}$ = 4.86 *NS*

ASAT = aspartate aminotransferase; ALAT = alanine amino-transferase; γGT = gamma glutamyl-transferase; Alkaline P = alkaline phosphatases; Total Chol. = total cholesterol.

Table S5. Comparison of enzymatic parameters of female rats of the 4 experimental groups injected intranasally with doses of aqueous solutions of AmyP53 of 0.2, 1.0 and 5.0 mg/kg BW every two days between D1 and D5, at D7 (n=3) (Mean ± SEM). Similar results were obtained with male rats.

Conclusion of the DRF study: For both male and female rats, no mortality, direct or induced, no signs of dysfunction, no body weight loss, no behavioral changes, no modification of food consumption, no significant modifications on almost all blood parameters measured, no alterations in the organs or tissues examined including the brain, and no abnormality of all the organs of the thoracic and abdominal cavities at autopsy were observed after repeated intranasal injections of doses of aqueous solutions of AmyP53 of 0.2, 1.0 and 5.0 mg/kg BW.

The maximal intranasal dose of AmyP53 without any signs of toxicity or dysfunction, following the treatment scheme used of one injection every two days during five days, is greater than or equal to 5.0 mg/kg BW in both male and female Wistar rats.
